# Microbiological Profile and Hygienic Quality of Foodstuffs Marketed in Collective Catering in Central Morocco

**DOI:** 10.1155/2023/2820506

**Published:** 2023-04-20

**Authors:** Rachid Amaiach, Sanae Lairini, Mouhcine Fadil, Moussa Benboubker, Rabia Bouslamti, Soukaina El Amrani, Abdelhakim El Ouali Lalami

**Affiliations:** ^1^Laboratory of Materials, Processes, Catalysis and Environment, University Sidi Mohamed Ben Abdellah, School of Technology, Post Office Box 2427, Fez, Morocco; ^2^Higher Institute of Nursing and Health Techniques of Fez, Regional Health Directorate Fez-Meknes, El Ghassani Hospital, 30000 Fez, Morocco; ^3^Physico-Chemical Laboratory of Inorganic and Organic Materials, Materials Science Center (MSC), Ecole Normale Supérieure, Mohammed V University in Rabat, Morocco; ^4^Human Pathology Bio-Health and Environment Laboratory, Faculty of Medicine and Pharmacy, Sidi Mohammed Ben Abdellah University, Fez, Morocco

## Abstract

Food hygiene is important both for its impact on the health of citizens and also for the cost of the infections that it can generate. In Morocco, it has become a concern of authorities. This work, realized for the first time in the center of Morocco, is aimed at describing the microbiological quality of foodstuffs marketed in collective catering in central Morocco. This study was conducted retrospectively from January 2015 to December 2019 in Fez city, central Morocco. The samples collected by the competent authority during official control from restaurants and food outlets were analyzed. Non-conformity was chosen as an indicator of food quality according to the official Moroccan standards. The samples were presented according to several variables: year/month/season, category/subcategory, communes, and establishment. The statistical processing of the results was done by SPSS 25. The Chi2 statistical test was calculated to determine a relationship between non-conformity and the type of analyzed matrix (year, season, and food category). The test was considered statistically significant for a *p* value < 0.05. A total of 2223 food samples were investigated, with an annual average of 445 samples. Overall, the rate of non-compliance during 2015-2019 was 31%, reaching its maximum in 2017 (36.4%) and its minimum in 2018 (27.5%).This rate varies by food type. Juices/drinks and meat products are the most contaminated with 71.7% and 58.1%, respectively, followed by milk and derivatives with 43.2%, seasoning sauces with 28.6%, pastries and pastry creams with 21.4%, and 14.4% for ready meals. The main causes of food non-conformity were fecal contamination germs with 67% positive fecal coliforms and 15% of total coliforms followed by total germs (7%), *Staphylococcus aureus* (5%), yeasts and molds (3%), sulfite-reducing anaerobes (2%), and *Salmonella* (1%). Given the obtained results, improving the hygienic quality of foods is necessary to ensure better consumer safety.

## 1. Introduction

Currently, food safety is a growing concern for all actors in the human food production chain. It plays a significant role in preventing foodborne diseases and consequently contributes to the control of health expenditures, as unsafe food represents a loss of revenue of around 110 billion dollars per year for low- and middle-income countries, due to productivity losses and related health costs [1].

In addition, contaminated food with pathogenic microorganisms such as bacteria can pose a serious risk to consumer health [2]. Therefore, it is essential to monitor the microbiological quality of foods to avoid the appearance of collective food poisoning (CFP).

Foodborne illnesses seem to be very common these days. Every day, more than 100, 000 people suffer from food poisoning. This gives sobering evidence. Certainly, the current food safety process is flawed and needs repair [3].

According to 2015 World Health Organization (WHO) estimates [1], foodborne hazards have caused 420,000 deaths. There are 600 million foodborne illnesses caused by pathogens such as non-typhoidal *Salmonella enterica*, *Salmonella* serotype Typhi (*S*. Typhi), and *Taenia solium,* to name but a few. The African region is the most vulnerable to foodborne diseases. There are over 91 million cases and 137,000 related deaths.

At the other extreme, in the European region, there are 23 million cases each year, of which 5000 are fatal. The majority of these cases are due to norovirus infections (about 15 million cases) and campylobacteriosis (5 million), but non-typical *Salmonella* causes the largest death rate (nearly 2,000 each year). Listeriosis also has severe health effects on those infected and causes around 400 deaths yearly; it is commonly transmitted through raw vegetables, ready-to-eat products, deli meats, smoked salmon, or soft cheeses [4].

In the United States and according to the Centers for Disease Control and Prevention (CDC) in 2011, approximately 48 million foodborne illnesses occur each year, including 128,000 hospitalizations and 3,000 deaths [3].

In France, nearly 2 million foodborne infections are reported each year. The most frequently suspected pathogens are *Salmonella*, *Staphylococcus aureus* (*S. aureus*), *Clostridium perfringens*, and *Bacillus cereus* [5].

In Morocco, foodborne illnesses have become a growing problem, both by their increasing frequency and the concern they arouse in the public opinion [6]. According to the Epidemic Diseases Department of the Directorate for Disease Control and Epidemiology, between 2008 and 2017, CFPs are frequent, and their incidence is gradually increasing over the years. The Ministry of Health reports around 1600 cases per year. However, the number is still underestimated, as many cases never reach the hospitals and are only reported when they worsen. Thus, we can estimate 10 cases for each declaration. The most frequently found are *Salmonella*, *Staphylococcus*, and fecal coliforms [7].

Data from the national epidemiological surveillance and health information system also show that 20-25% of the food establishments in the catering and retail sectors inspected by the health services are at risk each year. In recognition of the importance of food safety, the Ministry of Health, through the national food hygiene program, has defined strategic guidelines in food-related health risk monitoring and assessment, which focus mainly on reinforcing the foodborne disease and food safety monitoring system, developing the food risk assessment process, and raising awareness of consumers and professionals on food risk prevention measures [8]. This will be done in collaboration with the National Food Safety Office (ONSSA) [9].

In this context, this study, realized for the first time in the center of Morocco, is aimed at describing the profile and evaluating the microbiological quality of foodstuffs marketed in central Morocco during the period 2015 to 2019. The results of this study will be an important contribution to the programs and to services of health, especially to reinforcing the foodborne disease and food safety monitoring system and raising awareness of consumers and professionals on food risk prevention measures especially in the center of Morocco.

## 2. Materials and Methods

### 2.1. Type, Period, and Design of the Study

This is a retrospective study covering the period from January 2015 to December 2019 of the analyzed food samples analyzed at the Regional Laboratory of Epidemiological Diagnosis and Environmental Hygiene of Fez (RLEDEHF), using the quarterly reports of the epidemiological surveillance of food hygiene, especially the results of the microbiological food analysis, and those of the foodstuff sanitary inspections. The RLEDEHF is certified according to the national NM ISO/CEI 17025 : 2005 [10], which is the highest recognized quality standard for testing and calibration laboratories; it establishes the general requirements of competency, impartiality, and consistency of laboratory activities.

### 2.2. Location of the Study

**Figure 1:**
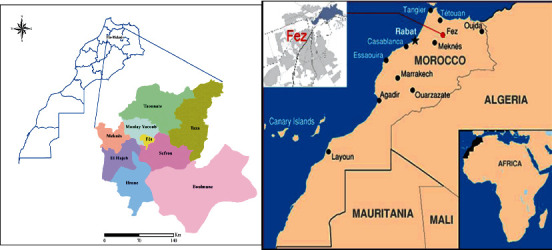
Geographic location of the study area (Fez Prefecture). Source: satellite location map of Fes, latitude/longitude: 34° 2′ 14^″^ N/4° 59′ 59^″^ W [13].

The Fez-Meknes region (Figure 1) covers an area of 40,075 km, i.e., 5.7% of the national territory and includes two administrative prefectures: the prefecture of Fez and the prefecture of Meknes. The area of the prefecture of Fez covers 332.1 km^2^. It is located in the plain of Saiss, halfway between the north and south of the Kingdom of Morocco, bounded on the north, northwest, and northeast by the province of Moulay Yacoub, southwest by the province of Sefrou. It is subdivided into (a) two urban communes: the commune of Mechouar Fez Jdid and the commune of Fez, which includes six urban districts (Agdal, Saiss, Zouagha, Mariniyéne, Fez Medina, and Jnane El Ward) and (b) three rural communes (Ouled Tayeb, Aïn Bida, and Sidi Harazem) (monograph of the region Fez-Meknes, 2015) [11, 12].

### 2.3. Sample Collection Sites

As part of the epidemiological surveillance, environmental health technicians carried out routine food sampling in restaurants and vulnerable food outlets in Fez.

Food samples were collected by health technicians and were transported to the laboratory in isothermal boxes equipped with cold accumulators and sterile bags at 0-4°C. Then, the samples were coded and recorded in a register; their analysis was carried out within six hours after their receipt.

Sampled products include meat, fish, fruits and vegetables, dairy products, salads, pastries, egg products, and ice cream made from milk. The analysis results have been sorted by category and subcategory for interpretation. A compliance assessment by food category allowed us to identify foods that are susceptible to microorganisms, which would require strict hygiene conditions during the handling.

### 2.4. Microbiological Analysis Methods

Laboratory technicians analyzed the food samples according to the official Moroccan procedures for microbiological analysis of foods [14, 15].

Germs were sought and counted according to predefined protocols; for total aerobic mesophilic flora (TAMF), the used plate count agar (PCA) was maintained in advance at a temperature of 47°C and cooled to 37°C. The plates were then incubated in an oven at 30°C. For total and fecal coliforms, the culture medium used was lactose deoxycholate agar (LDA); the Petri dishes were incubated for 24 hours at 37°C (for total coliforms) and 44°C (for fecal coliforms).

For *Salmonella*, the research is done in several steps: (i) nonselective preenrichment. The stock solution is incubated for 24 hours at 37° C and then used for the search for *Salmonella*. (ii) Enrichment phase in a liquid selective medium, which is Rappaport Vassiliadis (RV), consists of transferring 0.1 ml of the stock solution in a tube containing 10 ml of RV medium after preenrichment. The RV tube is incubated at 42°C and 37°C for 18 to 24 hours. Then (iii), isolation steps on a solid selective medium, which is Hektoen agar. A drop of enriched solution from the RV culture is streaked on a Petri dish previously poured with Hektoen agar. The plates are then incubated at 37°C for 18 to 24 hours.

When looking for *S. aureus*, Baird Parker agar was used, and the incubation was performed at 37°C for 24 hours. For sulfite-reducing anaerobes (SRA), sulfite polymyxin sulfadiazine agar (SPS) was used, and the incubation was performed at 46°C for 24 hours.

For yeast and mold, the preparations were inoculated on the surface of Sabouraud agar, initially prepared gentamicin and poured into Petri dishes, and incubated for 24 hours at 37°C. For detecting *Pseudomonas aeruginosa* (*P. aeruginosa*), the cetrimide agar was used and incubated at 36°C for 21 to 44 hours. It should be noted that the detection of *P. aeruginosa* must be followed by orientation tests: oxidase test and Gram stain to presume *P. aeruginosa*, and then biochemical tests for confirmation.

The microbiological analysis results obtained were interpreted according to Moroccan regulatory standards [14, 15]. We have retained the non-conformity event (NC) to the recommended standards as an indicator of food quality and subsequently, of poor hygienic quality.

### 2.5. Data Analysis

Statistical analysis was performed with the SPSS version 25 and Excel 19 to classify the data and make graphs. The data have been presented in tables, figures, and graphs to facilitate their interpretation.

The Chi2 statistical test was calculated to determine a relationship between non-conformity and the type of analyzed matrix (year, season, and food category). The test was considered statistically significant for a *p* value < 0.05.

## 3. Results

A total of 2223 food samples were collected during five years (more than 400 samples per year). The results of this study are distributed below by time, space, and hygienic quality of foods analyzed at the LRDEHMF from 2015 to 2019.

The non-compliance rate of foodstuffs analyzed according to their temporal and spatial distribution and category is shown in Table 1.The difference was statistically significant between separate years (*χ*2 = 70.84, *p* value < 0.001), months (*χ*2 = 193.85, *p* value < 0.001), seasons (*χ*2 = 80.58, *p* value < 0.001), communes (*χ*2 = 4941.61, *p* value < 0.001), categories (*χ*2 = 2366.31, *p* value < 0.001), and subcategories (*χ*2 = 2564, 58, *p* value < 0.001).

The Chi2 test confirms that there is a statistically significant association between the non-compliance and, respectively, years (*χ*2 = 11.927, *p* value = 0.018), months (*χ*2 = 49.052, *p* value < 0.001), season (*χ*2 = 16.20, *p* value < 0.001), communes of the city of Fez (*χ*2 = 18.08, *p* value < 0.001), food category (*χ*2 = 260.81; *p* value < 0.001), and food subcategories (*χ*2 = 466, 51, *p* value < 0.001).

According to this cross-tabulation (Table 1), it can be noticed that the number of samples analyzed has gradually decreased during the five years going from 532 samples in 2015 to 320 samples in 2019 with an average of 444.6, the total number of samples processed being 2223.

The non-compliance rate varies slightly from year to year; it increased from 2015 to 2017 and then decreased in 2018 to increase in 2019. The non-compliance rate reached its maximum value in 2017 and its minimum value in 2018. The average non-conformity over the five years is 31%.

According to Table 1, it can be deduced that the non-conformity is higher in summer and autumn compared to the other seasons; on the other hand, the conformity rate is successively increased in the winter (71.8%) and spring (73.1%). The average non-conformity during the four seasons is 31%.

The highest monthly non-compliance rates have been reported in October (46.0%), August (41.1%), and July (36.6%).

Concerning the communes of the prefecture of Fez, a high rate of non-conformity has been observed in the rural commune of Sidi Harazem (38.9%) than in the urban commune of Mechouar Fez Jdid (38.2%), the urban commune of Fez (31.8%), and then after the rural commune Ouled Taib. The number of analyzed samples in these communes was 18, 55, 1988, and 162 for Sidi Harazem, Mechouar, Fez, and Ouled Taib, respectively.

This cross-tabulation also shows that non-compliance is mainly found in the category of juices and drinks (71.7%) than the meat and meat products (58.1%), milk and its derivatives (43.2%), vegetables and raw vegetables (36.6%), seasoning sauce (28.6%), pastries and pastry products (21.4%), and prepared dishes (14.4%).

According to Table 1, we can say that the major part of non-conformity is found in the subcategory juices and drinks (71.7%) than raw meat (59.7%), milk in bulk, lben and traditional raib (48.7%), milk-based ice cream (40.5%), vegetables and fruits (36.6%), ketchup/mayonnaise (28.6%), and pastries (21.3%). Others are 100% compliant, namely, hard cheese, pasteurized milk, raw butter, smoked salmon, cooked charcuterie, fresh shellfish, Yogo, milk powder, and fish-based ready meals.

**Figure 2:**
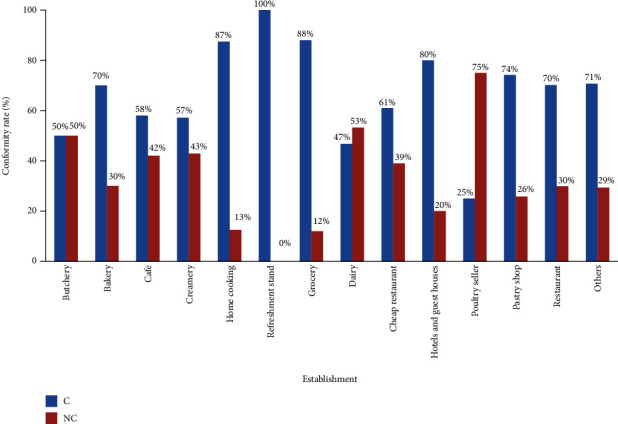
Conformity/non-conformity rate by establishment.

Concerning the non-compliance according to the type of establishment (Figure 2), the difference was statistically significant between food establishments (*χ*2 = 8863.11, *p* value 0.001).

Based on these data, we deduce that non-conformity is observed mainly at the level of poultry sellers (75%) then dairies (53%), butcheries (50%), and creameries (43%).

The Chi2 test confirms that there is a statistically significant relationship between food establishments and non-compliance (*χ*2 = 144.39, *p* value < 0.001). This means that the type of establishment impacts the rate of contamination.

**Figure 3:**
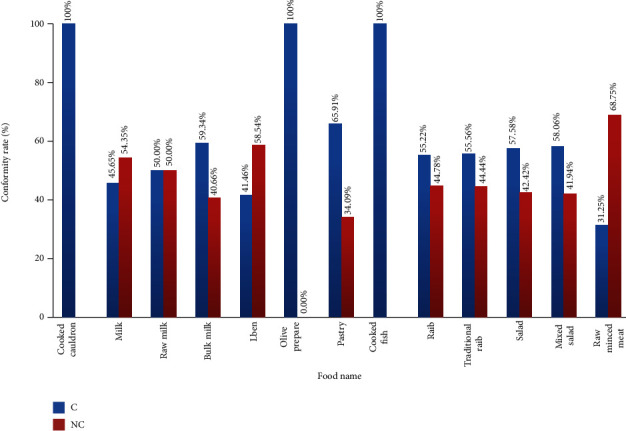
Conformity/non-conformity rate according to the food denomination.

Concerning the non-compliance according to the food denomination, Figure 3 shows that raw minced meat has the largest proportion of non-conformity (68.75%), lben (58.54%), milk (54.35%), and raw milk (50%), followed by raib (44.78%), traditional raib (44.44%), and bulk milk (40.66%).

**Figure 4:**
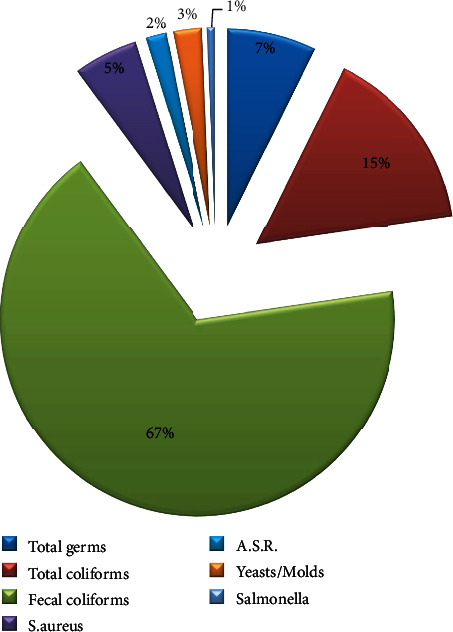
Non-conformity according to the germs.

As shown in Figure 4, we notice that the non-conformity according to the microorganisms is distributed as follows: total coliforms (67%) then fecal coliforms (15%), total germs (7%), *S. aureus* (5%), yeasts and molds (3%), A.S.R (2%), and *Salmonella* (1%).

Concerning the non-compliance for each food category by germ (Table 2), the Chi2 test confirms that there is a statistically significant association between the categories and, respectively, total germs (76.64, *p* value < 0.001), total coliforms (156.76, *p* value < 0.001), fecal coliforms (228.84, *p* value < 0.001), *S. aureus* (122.39, *p* value < 0.001), S.R.A. (70.21, *p* value < 0.001), yeast/mold (179.90, *p* value < 0.001), and *Salmonella* (22.23, *p* value < 0.004).

Table 2 shows that almost all the categories are contaminated most by fecal coliforms except category 6, which is not contaminated by any germ. This unusual result could be justified by the fact that for this food category, the 12 products sampled (Table 1) were collected cooked, and as a result, temperature lowered the number of bacteria below the non-compliance limits [14, 15].

The juice and drink categories are the most noncompliant, especially for coliforms, mold and yeast, and *S. aureus*.

### 3.1. Presentation of Results for Restaurants

According to the years, the rate of non-conformity increased in 2016 and then decreased in 2017 and 2018, and a peak of 33.3% was noted in 2019 (Table 3). We also found that the non-compliance rate increased in summer and autumn.

This cross-tabulation (Table 3) also shows that non-compliance is largely found in the category of meat and meat products (78.8%) than the seasoning sauce (75.0%), then vegetables and raw vegetables (36.2%), juices and drinks (33.3%), milk and its derivatives (21.4%), pastries and pastry products (19.0%), and prepared dishes (18.2%).

From Table 3, we can see that the food subcategory with a high rate of non-conformity is raw meat (77.8%), ketchup/mayonnaise (75%), milk-based ice cream (50%), vegetables/fruit (36.2%), juices and drinks (33.3%), then meat-based ready meals (25%), vegetable-based ready meals (22.4%) and pastries (19%).

The Chi2 test confirms that there is a statistically significant relationship between food subcategories and the occurrence of contaminations (*χ*2 = 348.010, *p* value < 0.001).

**Figure 5:**
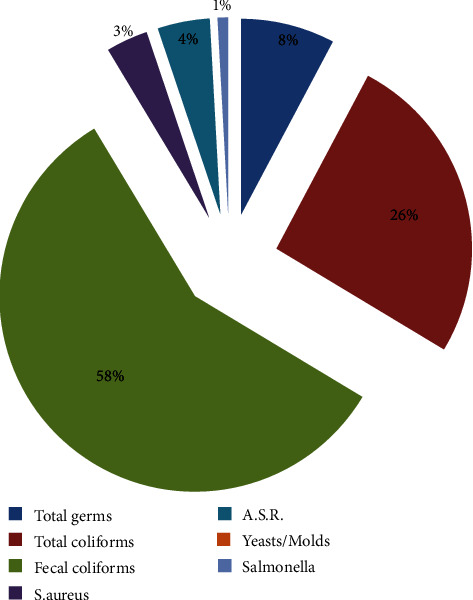
Non-conformity according to the germs.

According to Figure 5, it can be seen that 58% of the contamination of food marketed in restaurants was due to fecal coliforms, then 26% to total coliforms, 8% to total germs, 4% for S.R.A., 3% to *S. aureus*, and 1% for *Salmonella*.

## 4. Discussion

Out of the 2223 samples studied, our study showed that the majority (2043) originates from the urban areas of Fez. This can be explained by the fact that out of 1150131 inhabitants of the prefecture of Fez, the urban area represents 98.23%, and the rural area is only 1.77% [11, 12].

In general, the rate of non-compliance with foodstuffs in the urban area of Fez was 32% while that of the rural area was 18.9%. This rate varies from one commune to another. It is high in the rural commune of Sidi Harazem (38.9%), the urban commune of Mechouar Fez Jdid (38.2%), and the urban commune of Fez (31.8%). The concerned authorities are therefore invited to increase the number of hygiene inspections in these areas.

The high rate of non-compliance during summer revealed in our study could be due to the increasing temperature or lack of hygiene; this corroborates the results found by Miguéis et al. [16], who indicated that during the summer season, foods classified as unacceptable or very dangerous have been observed, which would require more accurate food safety systems to be put in place during the hot season.

In this region of Morocco, the very hot season extends from June 23 to September 10, with an average daily maximum temperature of over 32°C. The hottest month of the year is July, with an average maximum temperature of 35°C and minimum of 20°C. The cool season is from November 18 to March 16, with an average daily maximum temperature below 20°C. The coldest month of the year in Fez is January, with an average minimum temperature of 6°C and maximum of 16°C [17].

The statistical processing of the data allowed us to better explain the findings in this study. Based on the results of our research, we found seven types of germs: total germs, total coliforms, fecal coliforms, *S. aureus*, S.R.A., yeast/mold, and *Salmonella*.

Out of a total of 2223 samples, 688 are nonconforming, which corresponds to a percentage of contamination of 31% during the five years. This high contamination may raise the alarm about the hygienic quality of the food served in the concerned communes. This percentage varies from one establishment to another; indeed, the highest level of non-compliance was observed in poultry dealers (75%), followed by dairies (53%), butcheries (50%), and creameries (43%).The role of inspections should be very important in raising awareness and implementing corrective measures [18, 19].

Our study showed that the type of food establishment was associated with the rate of contamination, which is in accordance with the results of the study conducted by Alves et al. [20] who found that the proportion of contamination varied according to the type of food service unit. The highest rate of contaminated samples was found in fishmongers, butcher shops/charcuteries, and pastry/bakery.

The germs found in our study were the following total coliforms and fecal coliforms (82%in total), showing contamination of fecal origin, which would be a sign of non-compliance with good hygiene practices. These results are consistent with those found by Cohen et al. [21] and Chaiba et al. [22]. Usually, the presence of coliforms in food indicates ineffective heat processing or contamination following processing (e.g., pasteurization of milk). They may also be an indicator of poor cleaning and/or disinfection of equipment [22].

The rate of non-compliance with *Salmonella* is very low in our samples. However, the high rate of fecal coliforms, whose survival in the environment is close to *Salmonella*, leads to a slight suspicion. This result could be attributed to the possible presence of *Salmonella* competitors and inhibitor germs that the conventional method is unable to detect [23–25]. These germs are mainly fecal coliforms, e.g., *Citrobacter* [26, 27].

Indeed, the research for *Salmonella* in food products, especially meat products, poses many problems; their low number and the presence in these foods of a relatively abundant competitive flora make their research difficult, and these bacteria can cause misdiagnosis if their active multiplication outnumbers that of *Salmonella*, thus masking their presence [28].

According to the category food, a rate of non-compliance has been observed in juices and drinks (71.7%) than in meat and meat products (58.1%), in particular the raw meat and milk and its derivatives (43.2%). These results are consistent with those of Legnani et al. [29], who found that the most contaminated foods were raw meats and raw vegetables. Contrary, our results are not entirely coherent with those of the study by El Marnissi et al. [30], which specified that the category of food most considered noncompliant was milk and its derivatives (68.5%).

This non-compliance could be the result of the non-respect for the conservation rules, temperature, and cleanliness of the storage areas. Subsequently, the establishment must apply rigorous hygiene measures concerning hand washing, disinfection of the premises, and better control during the preparation, conservation, and delivery of these products.

In addition, the misapplications during the harvesting process at the slaughterhouse or on the farm have a negative impact on the microbiological quality of the animal product. To remedy this, (a) veterinary action on the farm must consist of the prevention and treatment of animal diseases and diseases that can be transmitted to humans (tuberculosis, brucellosis, etc.), and (b) at the slaughterhouse, it is necessary to control the establishment, its facilities, its operation, and the general hygiene of the building and the personnel and systematically control the animal before and after its slaughter [31].

Concerning the non-compliance for each food category by germs, our analysis revealed that the pastries and pastry creams were noncompliant for coliforms, total germs, and *S. aureus*. This is consistent with the study's finding that cream-filled pastries recovered from confectioneries are highly contaminated with foodborne pathogens and spoilage bacteria, including *Escherichia coli* (*E.coli*) (32.25%), coliforms (75.8%), and *S. aureus* (87.09%) [32], and also, Jamshidi et al. found that the contamination of semidried cream pastries by *E. coli* (coliform) was 43.2% [33].

Regarding the vegetables and raw vegetables, they were most contaminated with coliforms and with *S. aureus*; this corroborates the results revealed by Pereira et al. [34], and this finding was probably due to their contamination by soil, environment, and via water irrigation. Also, they can be altered during harvesting, transport, and processing consumption [27]. In addition, inadequate hand washing and subsequent handling of food could be a source of food contamination [34]. According to the WHO, many factors are the source of microbial contamination in production systems, particularly water quality, postharvest practices, workers' health, and hygiene and fertilizers [35].

Also, these values of coliforms, including *E. coli* observed in raw vegetables, would highlight an inadequate washing [36]. Furthermore, fecal coliforms were identified in 55% of fruits and vegetables [37]. This could present a potential health risk to consumers. Sanitation and personal hygiene need to be improved, especially during food processing.

In addition, the results found in this category suggest the need to employ strict control measures and develop preventive strategies to improve the quality and safety of fresh fruits and vegetables for consumption [38–41].

For meat and meat products, the category was contaminated by all germs, especially by fecal coliforms except for total coliforms. Several sources can interfere with the microbiological quality of meat products, such as the preservation methods applied [42]. Several studies have revealed that poultry meat is mainly contaminated by *Salmonella* [43, 44]. This may be due to poultry meat refining methods where *Salmonella* is abundant in this processing environment [45].

For milk and milk products like milk in bulk, lben, and traditional raib, it was contaminated especially by *S. aureus* and to a lesser extent by coliforms, sulfite-reducing anaerobic bacteria, and *Salmonella*, which is consistent with the results of Belomaria et al. who found that leading causes of food poisoning in Morocco are fruits and vegetables (20%) and dairy products (17%), and the causative germs are *S. aureus* (72%) and *Clostridium perfringens* (28%) [7].

According to Little et al., raw milk was of unsatisfactory quality in cheese preparation due to contamination by *S. aureus*, *E. coli*, and *Listeria monocytogenes* [46]. Furthermore, according to Organji et al. [47], raw milk ready to drink has low microbiological quality, and its consumption should be avoided.

For juices and drinks, this category was highly contaminated by the germs studied, except for S.R.A. and *Salmonella*, which would require particular attention to the hygienic preparation of these foodstuffs.

On the other hand, fish and fish products were compliant, but caution should be applied to this type of foodstuff since the data proved its contamination [48].

Moreover, contamination in restaurants had a high level of non-compliance (28.6%); the foodstuffs concerned were raw meat, ketchup and mayonnaise, milk-based ice cream, vegetables/fruits, meat-based ready meals, and vegetable-based ready meals, which indicates non-compliance with hygiene rules and non-application of HACCP (hazard analysis critical control point) [49]. This is in line with the study conducted in catering establishments by Kadmiri et al. [18]. WHO stated that the non-respect of these rules, in particular the inappropriate storage temperatures, the mixing of hot and cold food, and the handling of food with dirty utensils and without gloves, exposes all the products to contamination. It can also be said that adequate and regular disinfection of food contact surfaces has not been achieved to reduce the risk of food contamination.

The researchers emphasize the importance of bacteriological surveillance of food in restaurants to ensure the absence of bacterial contamination in the food offered to customers, especially ready-to-eat foods [50–53], and to control the food preparation chain. The production of high-quality microbiological meals requires compliance with hygiene rules at several levels; raw materials used preparation environment (equipment, storage, premises, and personnel) to prevent the occurrence of collective food poisoning in restaurants.

Given the findings from this study focusing on the microbiological quality of foodstuffs marketed in the restaurants of Fez and the profile of bacteria isolated, we thought it would be useful to offer suggestions that could help to reduce the prevalence of their spread, to keep the hygienic quality of foodstuffs high, and to minimize the diseases resulting from contamination.

The following recommendations are of relevance to the establishments covered by the study, including restaurants: (a) adopt an integrated management of inspection activities in food establishments; (b) implement a quality approach in food establishments not only in order to guarantee quality and food hygiene but also to increase traceability throughout the food chain; (c) conduct an effective training on the principles of hygiene for the personnel of the kitchen [54]; (d) check that the information provided in the HACCP program are applied [55, 56]; (e) improve the cleaning system during the manufacturing process: floor, walls, cold rooms, toilets, and devices; (f) mandatory hand washing after any contaminating activity; (g) establish protocols for monitoring and controlling humidity and temperature in vegetable storage areas; (h) replace manually operated faucets and doors to the cooking area with automatic or operable ones; and (i) serve food immediately and following cooking, and cooked food should be stored in an appropriate container protected from contamination.

Finally, improvements and awareness programs on the importance of hygienic quality can be made; protocols and procedures must be written, validated, and regularly evaluated to fight against foodborne health problems, i.e., foodborne diseases.

## 5. Conclusion

Our study showed a high percentage of contamination of foodstuffs marketed in collective catering, and the most affected categories of foodstuffs are meat and meat products, juices and drinks, and milk and its derivatives. This high level of contamination would suggest a lack of compliance with hygiene rules during food preparation, storage, and distribution to consumers. The non-compliance was specifically due to the remarkable presence of fecal coliforms, yeast and molds, sulfite-reducing anaerobes, and *S. aureus*. This microbiological ecology shows a deficiency in hygiene measures, especially when handling and processing the raw material of foodstuffs.

Given the results, there is a need to raise awareness among staff working in food establishments, especially those handling food, and the need to respect hygiene rules to ensure that the consumer receives not only good but also healthy food, thus avoiding cases of food poisoning. As a prospect, it would also be beneficial to study the related factors of microbiological non-compliance and hazard analysis to demonstrate adequate control.

## Figures and Tables

**Table 1:** Percentage and frequency of non-compliance of foodstuffs analyzed during the study period.

Distribution of foodstuffs	Samples *n* (%^∗^)	Conformity	
		Compliant *n* (%^∗∗^)	Non-compliant *n* (%^∗∗^)
Year			
(i) 2015	532 (23.9)	383 (72)	149 (28)
(ii) 2016	500 (22.5)	338 (67.6)	162 (32.4)
(iii) 2017	486 (21.9)	309 (63.6)	177 (36.4)
(iv) 2018	385 (17.3)	279 (72.5)	106 (27.5)
(v) 2019	320 (14.4)	226 (70.6)	94 (29.4)
Total	2223 (100)	1535 (69)	688 (31)
Season			
(i) Autumn	588 (26.5)	377 (64.1)	211 (35.9)
(ii) Summer	375 (16.9)	245 (65.3)	130 (34.7)
(iii) Winter	625 (28.1)	449 (71.8)	176 (28.2)
(iv) Spring	635 (28.6)	464 (73.1)	171 (26.9)
Total	2223 (100)	1535 (69)	688 (31)
Commune			
(i) Urban	2043 (92)	1389 (68)	654 (32)
(ii) rural	180 (8)	146 (81.1)	34 (18.9)
Total	2223(100)	1535 (69)	688 (31)
(i) Urban community of Fez	1988 (89.4)	1355 (68.2)	633 (31.8)
(ii) Urban community of Mechouar	55 (2.5)	34 (61.8)	21 (38.2)
(iii) Rural community of Ouled Taib	162 (7.3)	135 (83.3)	27 (16.7)
(iv) Rural community of Sidi Harazem	18 (0.8)	11 (61.1)	7 (38.9)
Total	2223 (100)	1535 (69)	688 (31)
Category			
(i) Meat and meat products	186 (8.3)	78 (41.9)	108 (58.1)
(ii) Juices and drinks	46 (2.1)	13 (28.3)	33 (71.7)
(iii) Milk and milk products	574 (25.9)	326 (56.8)	248 (43.2)
(iv) Vegetables and raw vegetables	331 (14.9)	210 (63.4)	121 (36.6)
(v) Seasoning sauces	28 (1.3)	20 (71.4)	8 (28.6)
(vi) Pastries and pastry creams	276 (12.4)	217 (78.6)	59 (21.4)
(vii) Prepared dishes	762 (34.3)	652 (85.6)	110 (14.4)
(viii) Eggs and egg products	8 (0.4)	7 (87.5)	1 (12.5)
(ix) Fish and fish products	12 (0.5)	12 (100)	0 (0)
Total	2223 (100)	1535 (69)	688 (31)
Subcategory			
(i) Raw meat	181 (8.1)	73 (40.3)	108 (59.7)
(ii) Milk in bulk, lben, traditional raib	468 (21)	240 (51.3)	228 (48.7)
(iii) Juices and drinks	46 (2)	13 (28.3)	33 (71.7)
(iv) Ready-made meals with vegetables	461 (20.7)	394 (85.5)	66 (14.3)
(v) Ready-made meals with meat	94 (4.2)	71 (75.5)	23 (24.5)
(vi) Prepared dishes based on poultry	140 (6.3)	122 (87.1)	18 (12.9)
(vii) Ready-made meals based on fish	57 (2.6)	57 (100)	0 (0)
(viii) Ketchup, mayonnaise	28 (1.2)	20 (71.4)	8 (28.6)
(ix) Vegetables and fruits	331 (14.9)	210 (63.4)	121(36.6)
(x) Egg products	8 (0.35)	7 (87.5)	1 (12.5)
(xi) Pastries	277 (12.4)	218 (78.7)	59 (21.3)
(xii) Soft cheese, Jben	41 (1.8)	36 (87.8)	5 (12.2)
(xiii) Ice cream made from milk	37 (1.6)	22 (59.5)	15 (40.5)
(xiv) Raw charcuterie	12 (0.5)	9 (75.0)	3 (25.0)
(xv) Cooked charcuterie	5 (0.2)	5 (100)	0 (0)
(xvi) Fresh shellfish	1 (0.04)	1 (100)	0 (0)
(xvii) Hard cheese	10 (0.4)	10 (100)	0 (0)
(xviii) Pasteurized milk	5 (0.2)	5 (100)	0 (0)
(xix) Fresh fish	8 (0.35)	8 (100)	0 (0)
(xx) Milk powder	1 (0.04)	1 (100)	0 (0)
(xxi) Raibi; Yogo; industrial milk	4 (0.2)	4 (100)	0 (0)
(xxii) Smoked salmon	1 (0.04)	1 (100)	0 (0)
(xxiii) Raw butter	7 (0.3)	7 (100)	0 (0)
Total	2223 (100)	1535 (69)	688 (31)

*n*: number of samples. ^∗^: within the total number of samples. ^∗∗^: within the distribution of foodstuffs.

**Table 2:** Percentage of non-compliance for each food category by germ.

Food category	Germs						
	Total germs (%^∗^)	Total coliform (%^∗^)	Fecal coliform (%^∗^)	*S. aureus* (%^∗^)	S.R.A. (%^∗^)	Yeast and mold (%^∗^)	*Salmonella* (%^∗^)
(1) Milk and milk derivatives	2.8	2.6	40.4	0.3	0	0	0.20
(2) Eggs and egg products	0	0	12.5	0	0	0	0
(3) Pastry and pastry creams	1.4	6.5	19.6	1.4	0	0	0
(4) Prepared dishes	2.0	4.9	13.1	0.4	0.1	0	0
(5) Vegetables and raw vegetables	2.4	11.8	34.4	1.2	0	0	0
(6) Meat and meat products	4.3	0	51.6	13.4	8.1	0.5	2.7
(7) Fish and fish products	0	0	0	0	0	0	0
(8) Seasoning sauces	0	28.6	10.7	0	0	0	0
(9) Juices and drinks	39.1	56.5	54.3	23.9	0	47.8	0

^∗^: within the food category.

**Table 3:** Percentage and frequency of non-compliance of foodstuffs from restaurants.

Distribution of foodstuffs	Samples *n* (%^∗^)	Conformity	
		Compliant *n* (%^∗∗^)	Non-compliant *n* (%^∗∗^)
Years			
(i) 2015	63 (22.5)	46 (73.0)	17 (27.0)
(ii) 2016	53 (18.9)	36 (67.9)	17 (32.1)
(iii) 2017	93 (33.2)	65 (69.9)	28 (30.1)
(iv) 2018	50 (17.9)	39 (78.0)	11 (22.0)
(v) 2019	21 (7.5)	14 (66.7)	7 (33.3)
Total	280 (100)	200 (71.4)	80 (28.6)
Seasons			
(i) Autumn	71 (25.6)	42 (59.2)	29 (40.8)
(ii) Summer	49 (17.5)	33 (67.3)	16 (32.7)
(iii) Winter	80 (28.5)	67 (83.8)	13 (16.3)
(iv) Spring	80 (28.5)	58 (72.5)	22 (27.5)
Total	280(100)	200 (71.4)	80 (28.6)
Category			
(i) Meat and meat products	27 (9.6)	6 (22.2)	21 (78.8)
(ii) Seasoning sauces	4 (1.4)	1 (25.0)	3 (75.0)
(iii) Vegetables and raw vegetables	58 (20.7)	37 (63.8)	21 (36.2)
(iv) Juices and drinks	3 (1.1)	2 (66.7)	1 (33.3)
(v) Milk and milk products	14 (5.0)	11 (78.6)	3 (21.4)
(vi) Pastries and pastry creams	21 (7.5)	17 (81.0)	4 (19.0)
(vii) Prepared dishes	148 (52.8)	121 (81.8)	27 (18.2)
(viii) Fish and fish products	5 (1.8)	5 (100)	0 (0)
Total	280 (100)	200 (71.4)	80 (28.6)
Subcategory			
(i) Raw meat	27 (9.6)	6 (22.2)	21 (77.8)
(ii) Ketchup, mayonnaise	4 (1.4)	1 (25)	3 (75)
(iii) Ice cream made from milk	6 (2.1)	3 (50)	3 (50)
(iv) Cooked meats	2 (0.7)	1 (50)	1 (50)
(v) Vegetables and fruits	58 (20.7)	37 (63.8)	21 (36.2)
(vi) Juices and drinks	3 (1.1)	2 (66.7)	1 (33.3)
(vii) Ready-made meals with meat	12 (4.3)	9 (75)	3 (25)
(viii) Ready-made meals with vegetables	85 (30.4)	66 (77.6)	19 (22.4)
(ix) Pastries	21 (7.5)	17 (81)	4 (19)
(x) Prepared dishes with poultry	33 (11.8)	29 (87.9)	4 (12.1)
(xi) Soft cheese, Jben	1 (0.4)	1 (100)	0 (0)
(xii) Hard cheese	5 (1.8)	5 (100)	0 (0)
(xiii) Milk in bulk, lben, traditional raib	2 (0.7)	2 (100)	0 (0)
(xiv) Ready-made meals based on fish	18 (6.4)	18 (100)	0 (0)
(xv) Fresh fish	3 (1.1)	3 (100)	0 (0)
Total	280 (100)	200 (71.4)	80 (28.6)

*n*: number of samples. ^∗^: within the total number of samples. ^∗∗^: within the distribution of foodstuffs.

## Data Availability

Data analyzed during this study are all included in the main manuscript.
